# Analysis of population structures of the microalga *Acutodesmus obliquus* during lipid production using multi-dimensional single-cell analysis

**DOI:** 10.1038/s41598-018-24638-y

**Published:** 2018-04-19

**Authors:** Michael Sandmann, Michaela Schafberg, Martin Lippold, Sascha Rohn

**Affiliations:** 1 Institute for Food and Environmental Research (ILU e. V.), Arthur-Scheunert-Allee 40-41, 14558 Nuthetal, Germany; 2 Hamburg School of Food Science, Institute of Food Chemistry, University Hamburg, Grindelallee 117, 20146 Hamburg, Germany

## Abstract

Microalgae bear a great potential to produce lipids for biodiesel, feed, or even food applications. To understand the still not well-known single-cell dynamics during lipid production in microalgae, a novel single-cell analytical technology was applied to study a well-established model experiment. Multidimensional single-cell dynamics were investigated with a non-supervised image analysis technique that utilizes data from epi-fluorescence microscopy. Reliability of this technique was successfully proven via reference analysis. The technique developed was used to determine cell size, chlorophyll amount, neutral lipid amount, and deriving properties on a single-cellular level in cultures of the biotechnologically promising alga *Acutodesmus obliquus*. The results illustrated a high correlation between cell size and chlorophyll amount, but a very low and dynamic correlation between cell size, lipid amount, and lipid density. During growth conditions under nitrogen starvation, cells with low chlorophyll content tend to start the lipid production first and the cell suspension differentiated in two subpopulations with significantly different lipid contents. Such quantitative characterization of single-cell dynamics of lipid synthesizing algae was done for the first time and the potential of such simple technology is highly relevant to other biotechnological applications and to deeper investigate the process of microalgal lipid accumulation.

## Introduction

An increasing world population, decreasing fossil fuel deposits, and the need for sustainable food and energy resources are the key drivers in microalgae research. The biochemical composition of the algal biomass can be modulated by varying growth conditions. As a consequence, the oil content can be elevated. In this context, the green alga *Acutodesmus obliquus* is able to accumulate up to 45% w/w triacylglycerol (TAG) content when grown under nitrogen starvation^1^. TAG yield can be used e.g., for edible oils, technical fats, or biodiesel. Yet, the formation of TAG in microalgae has not been fully understood. It is widely accepted that the absorption of photons via pigments in the photosynthetic apparatus can easily generate excess energy in the cells. This is valid especially under stress conditions. The cells channel this excess energy from light into storage compounds such as starch and/or TAG^2–4^. Correspondingly, the formation of reactive oxygen species is minimized^5^. Substantial work was done in nitrogen starvation experiments aiming at stimulating some microalgae species to generate a high lipid content^6,7^. Lipid amount in algal cells is usually quantified by traditional solvent extraction procedures, followed by fractionation and gravimetric estimation^8,9^. Afterwards, HPLC or GC analysis can be used to characterize the fatty acid composition of the lipids. Besides others, these common analytical technologies deliver a mean value over large sample sets with high numbers of cells and each conclusion drawn out of this analysis is based on the assumption of a homogenous lipid distribution and cell population. During the last years, single-cell-analytical techniques (SCA) became an emerging field in biology and medicine^10–12^. Those approaches, based on different methods, are capable of revealing heterogeneity in cell populations that are not accessible with bulk methods. In biotechnology, understanding the dynamics of heterogeneity in bioprocesses is considered to be the key for higher productivity and product quality^12–15^. Cell-to-cell heterogeneity might have a significant impact on the productivity of bioprocesses and on product quality^16^. Thus, the quantification of the heterogeneity is recently recognized as a tool for bioprocess description and optimization with great potential for future applications^12^. Probably the most common technique in this field is flow cytometry (FC) in which fluorescent properties and light scattering intensity at different angles of cells can be analyzed^17^. Use of FC in the field of algae has been recently reviewed^18^. A deep insight of the technical advantages and disadvantages of FC is given by Shapiro (2003)^17^. In addition to FC, microscopy-based single-cell analysis and automated object recognition became an emerging tool for SCA^19–22^. The advantages of this so-called microscopic cytometry in comparison to FC, are direct access to cellular size and morphological properties that are intrinsically embedded within the microscopic images and the access on spatial information enabling subcellular analysis. Additionally, time laps experiments can be done, directly on specialized microscope slides, to follow dynamics of single cells over longer periods^21^. In this interdisciplinary field, a parallel development of advanced microscopic setups, their use in bioanalytical research, and of novel object recognition algorithms occurred. Algorithms for automated object recognition are strongly beneficial for quantitative analysis of large sample sets. They are further needed for standardized protocols for deciding how the complex cellular information has to be extracted from the images. Despite the more and more extensive use of single-cell analytical technologies in medicine, these technologies are not yet established as tools to quantitatively describe multidimensional single-cell dynamics in systems biology and biotechnology. One of the first studies in the field of algal biology was the usage of a second harmonic generation (SHG) microscopy approach to quantitatively describe the starch metabolism on the level of single cells of *Chlamydomonas*^23^. The laser scanning imaging technique applied was combined with an object recognition algorithm to extract information from 3-dimensional spatial information of cells during algal development. Based on this data, Rading *et al*. investigated the stochastic nature of these dynamics in a theoretical approach^24^. Sandmann *et al*. used this label-free microscopic technique to compare a mutant cell line with its wild-type background^25^. A very advanced label-free *in vivo* analysis of intracellular lipid droplets in algal cells by *Coherent Anti-Stokes Raman* Scattering (CARS) microscopy was recently established^26,27^. In those studies, complex microscopic setups with multiple lasers and time-gated detectors were used to discriminate between the Raman signals and the fluorescent background of the cells and the trajectory of lipid droplet formation was followed over time.

The structure of cell populations, meaning the phenotypic characteristics of various cells in a cell population, is dependent on multiple parameters including cultivation conditions. To the best of our knowledge, single-cell analytical technologies were not used before to quantitatively study the dynamics between chlorophyll, lipids, and the relation of these two fundamental cellular ingredients to population structures of algae before. In the presented work a simple and fast procedure for multi-dimensional single-cell analysis, based on epi-fluorescence microscopy and automated cell recognition, was developed for advanced process monitoring.

## Results

### Single-cell approach and microscopic images

**Figure 1:**
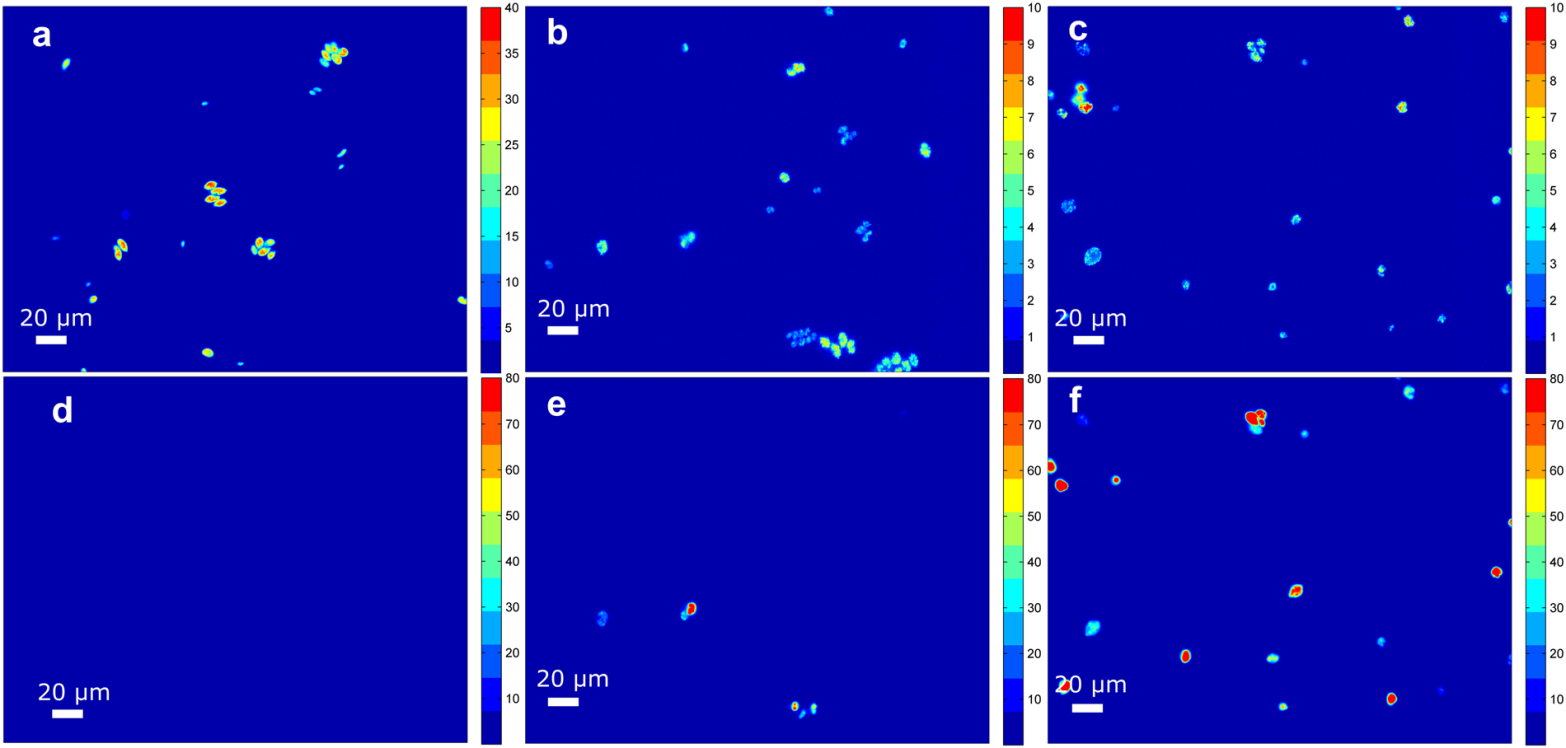
Microscopic images of the nitrogen starved culture (−N) coding chlorophyll and Nile Red (NR) fluorescence intensity. (**a**–**f**) False color imaging. Red: high signal intensity (high level of chlorophyll and TAGs); blue: low signal intensity (low level of chlorophyll and TAGs). (**a**–**c**) Chlorophyll fluorescence at day 0, day 4, and day 8, respectively. (**d**–**f**) NR fluorescence at day 0, day 4, and day 8, respectively.

Cells of the green alga *Acutodesmus obliquus* were exposed to nitrogen starvation and the response of the cells was investigated at the level of single cells. Experiments with nitrogen starvation have been conducted in basic research as model experiments for decades, as it is one of the most important strategies to increase triacylglycerol (TAG) content for e.g., biodiesel production. The microscopic approach is based on the automated extraction of the information from digital images derived from epi-fluorescent microscopy. Intrinsic pigment fluorescence of the cells and the well-established Nile Red (NR) staining was used to quantify chlorophyll and TAG content with single-cell resolution. The custom-designed algorithm used pigment fluorescence from the chloroplast of the algal cells as a trigger signal to detect the objects within the images. Cell size determination was conducted after image processing with an up-stream contrast enhancement method. This procedure artificially increased the light intensity that is scattered inside the non-fluorescent cell volume and thus, enables a proper determination of the cell size^19,23^. Following contrast enhancement, the discrimination between objects and background was done with a single threshold procedure combined with an optimization step to define the threshold^28^. Following pixel assignment to distinct cells, readout of pixel information from unmodified raw data was done for determining chlorophyll and TAG amount, respectively. A schematic drawing of the procedure is given in Supplementary Fig. S1. With this procedure, structures of cell populations can be characterized over time. Figure 1 shows representative images of the cells, illustrating how fluorescent signals derived from chlorophyll (Fig. 1a–c) and from NR (Fig. 1d–f) are changing. On the single-cell level, the cells showed high heterogeneity leading to a broad chlorophyll and TAG distribution at each time point during cultivation. The signal intensity per unit area (e.g., per pixel) also exhibited strong variability between the cells. Almost no lipid bodies (LB) were visible under control conditions and at the beginning of the experiment (Fig. 1d). During nitrogen starvation, the cells tended to synthetize LB, which mainly contain TAG. This formation already started in some cells after one day. At the end of the experiment, TAG content increased strongly (Fig. 1f) and a high proportion of cells were filled to a large extent with LB. Another known effect, related to nitrogen starvation (-N), is degradation of the chloroplast and its pigments, to prevent over-excitation of the photosynthetic apparatus during stress. Over time, the chlorophyll fluorescence coding the chlorophyll content per chloroplast decreased strongly (Fig. 1a–c) and the ellipsoidal chloroplast turned into a more sponge-like structure (please note the changed heat map scale for better illustration in Fig. 1a–c).

### Development of cells

**Figure 2:**
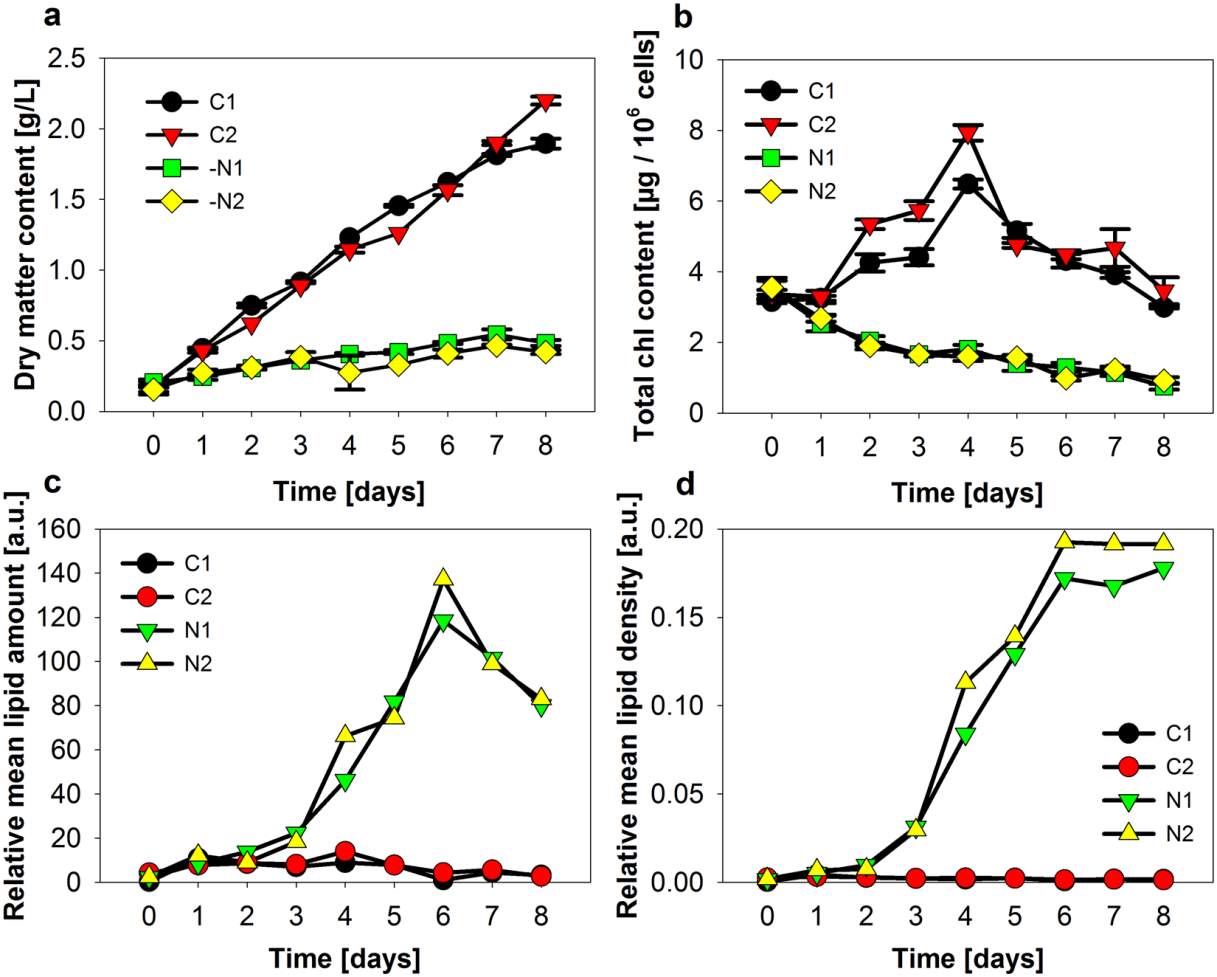
Biomass production, pigment, and TAG content in cultures of *Acutodesmus o*. (**a**) Dry matter content per suspension volume, (**b**) Chlorophyll content per one million cells, (**c**) relative mean TAG amount, and (**d**) relative mean TAG density are given. Means of 2 technical replica ± S.D. for (**a**) and 3 technical replica for (**b**). For (**c**,**d**) averages from single-cell datasets are given. Between 305 and 1012 cells were included in the particular datasets.

Growth, photosynthetic apparatus, and the pigment content was strongly affected by nitrogen starvation, which is represented by a decrease of the chlorophyll content within the cells. Mean growth rate was 0.23 g/ L*d and 0.03 g/ L*d, for the control and the -N-culture, respectively (Fig. 2a). Total chlorophyll content, based on one million cells, primarily increased until halftime of the experiment, followed by a decrease for the control culture (Fig. 2b), whereas chlorophyll in the -N culture decreased continuously over time. Both cultures differed strongly in the development of mean TAG content, which was determined with the microscopic technique (Fig. 2c,d) through averaging single-cell data from a large sample set of cells. For the -N-culture, Lipid amount per cell reached a maximum at day six (Fig. 2c). The measured TAG density, which can be interpreted as TAG concentration, showed an approx. 100-fold increase. TAG content was comparatively constant for the control cultures.

### Validation of the microscopic approach

The validity of the interplay between the chosen microscopic setup and the object recognition algorithm was proven with a typical reference analysis. Supplementary Fig. S2 shows the total chlorophyll content per cell over the duration of the experiment. Data from traditional solvent extractions were taken as a reference (Fig. 2b). For better comparability of both methods, datasets were transformed into a relative scale by dividing all values from a particular timepoint by the mean value obtained from the dataset. The test for linear correlation resulted in an R^2^ of 0.959 for the control and R^2^ of 0.891 for the -N-dataset, which corresponded to a linear dependency between both quantification techniques. It was shown in various studies that the NR staining and determination of the TAG content by fluorescence detection is in good agreement to neutral lipid determination with reference analysis^29–32^. This also holds true for different algal cells, emulsions, and animal cell lines^29,33,34^. In the present work, another strategy was chosen and a comparison between microscopy and fluorescence quantification by a spectrometer was done. Cells under nitrogen-starved conditions and with high TAG content were stained with six different NR concentrations and the signal dependency was followed with both techniques (Supplementary Fig. S2). A linear correlation with a R^2^ of 0.963 was observed. Additionally, reliability of the object recognition capabilities of the algorithm was recently proven with different particle size standards and with cells from *A. obliquus*^19^. Some physical phenomena can disturb the optical analysis of TAG and chlorophyll in algal cells. One of the most discussed disadvantage of algal cells is the autofluorescence, potentially overlapping with the lipid/fatty acid signals^18,27,29,35^. For minimisation of disturbing effects, the use of spectral filters enabling separation of the illuminating photons from the emitted photons is necessary^17^. Supplementary Fig. S2 shows fluorescence emission spectra derived from non-stained and NR-stained cells of *A. obliquus* grown under nitrogen starvation. Additionally, spectral ranges of the used filter sets for excitation and emission are shown. The microscopically determined amount of background fluorescence in the spectral range for the NR detection was around 6% of the positive signal with applied NR staining (final concentration of 2 µg/mL) (Supplementary Fig. S2). The negligibility of the potential background fluorescence in the NR detection range can be also seen in the microscopic image in Fig. 1d. Within the high dynamic range of the cellular development, the cells do not exhibit ether a significant NR signal nor a background fluorescence (Fig. 1d). The second potentially disturbing phenomenon might be energy transfer from excited NR to chlorophyll. In this case photons emitted by NR could be reabsorbed by the chlorophyll molecules causing a decrease of NR-fluorescence and an increase of chlorophyll fluorescence. As chlorophyll is an intrinsic chromophore, simple experimental controls proving this situation are not possible. Nevertheless, also this energy transfer path seems to be not significant in this experimental setup. This can be concluded, from the opposed development of the strong increase of NR fluorescence intensity over time and the decrease of chlorophyll fluorescence in the -N-cultures (Fig. 2 and Supplementary Fig. S2). In this situation, a possible energy transfer from NR to chlorophyll could be easily promoted. If so, a clear overestimation of chlorophyll content for the later time points should be determined by the microscopic technique in comparison to the reference technique. This was not found to be valid (Supplementary Fig. S2). On basis of the described optical effects a slight over or underestimation of the cellular ingredients could occur, but it was shown that these deviations are not relevant to the dynamics described in the experiments. Based on the data of the present study, the microscopic technique was able to detect objects from images and to determine cellular constituents for cells under nitrogen starvation or control conditions.

### Description of population structures: Multidimensional analysis

**Figure 3:**
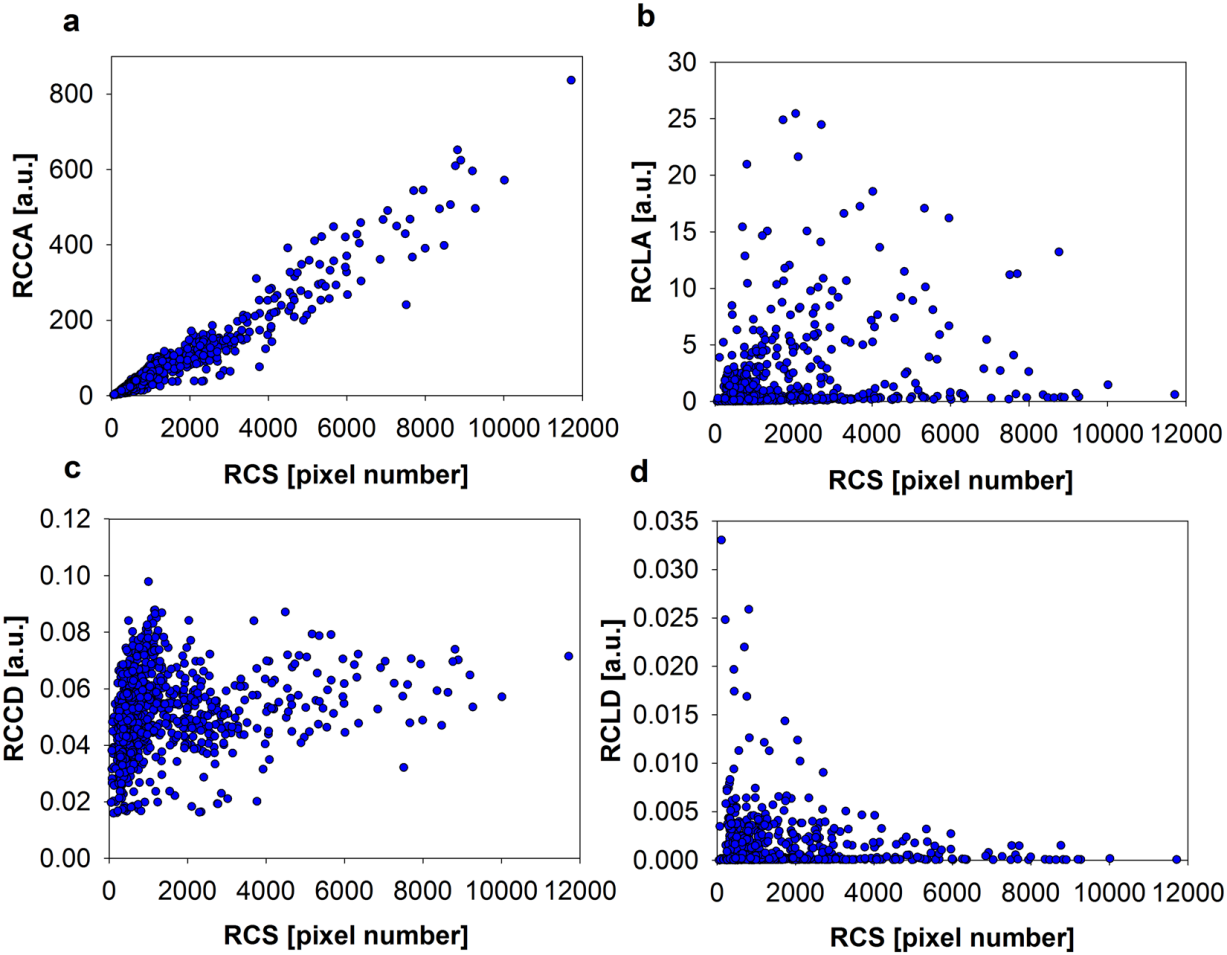
Scatter plots of the cellular chlorophyll and lipid amount as determined by single-cell analysis at day 0. (**a**) Relative cellular chlorophyll amount (RCCA), (**b**) relative cellular lipid amount (RCLA), (**c**) relative cellular chlorophyll density (RCCD), and (**d**) relative cellular lipid density (RCLD). Each dot represents one characterized cell (analyzed cell number is n = 1008). Cell size is given as primary data in pixel number.

The major advantage of the microscopic cytometry is the ability to perform a multidimensional analysis, with direct access on cell size information, which is intrinsically encoded in the microscopic images of the objects. Scatter plots visualize dependencies between different properties on a single-cellular level (Fig. 3). Each dot in the diagrams represents a single cell. *Relative cellular chlorophyll amount* (RCCA), determined by fluorescence microscopy showed a relatively linear dependency with *relative cell size* (RCS) with a Spearman’s rank correlation coefficient (Spearman rho; test for general correlation) of 0.96 (Supplementary Table S1 and 2). Spearman rho of the other samples showed similar values, slightly below 1. Despite, a relatively high heterogeneity was found in the RCCA in relation to a given RCS. This can be visualized by the *relative cellular chlorophyll density* (RCCD), which can be interpreted as chlorophyll concentration (Fig. 3c). However, the relationship of the *relative cellular lipid amount* (RCLA) against the corresponding RCS was different. The scatter cloud exhibited no clear dependency (Fig. 3b), with very small Spearman rho around zero for the whole experiment (Supplementary Table S1 and 2). The *relative cellular lipid density* (RCLD) exhibited a very different shape indicating that small cells possess a relatively high RCLD and very large cells contain a relatively low RCLD. On the single-cell level, the correlation between RCS and RCLD was very low throughout the whole experiment (Supplementary Table S1 and 2) and all parameters measured exhibited a high heterogeneity in the cell suspension.

### Description of population structures: Development of lipid content and characterization of occurring subpopulations

**Figure 4:**
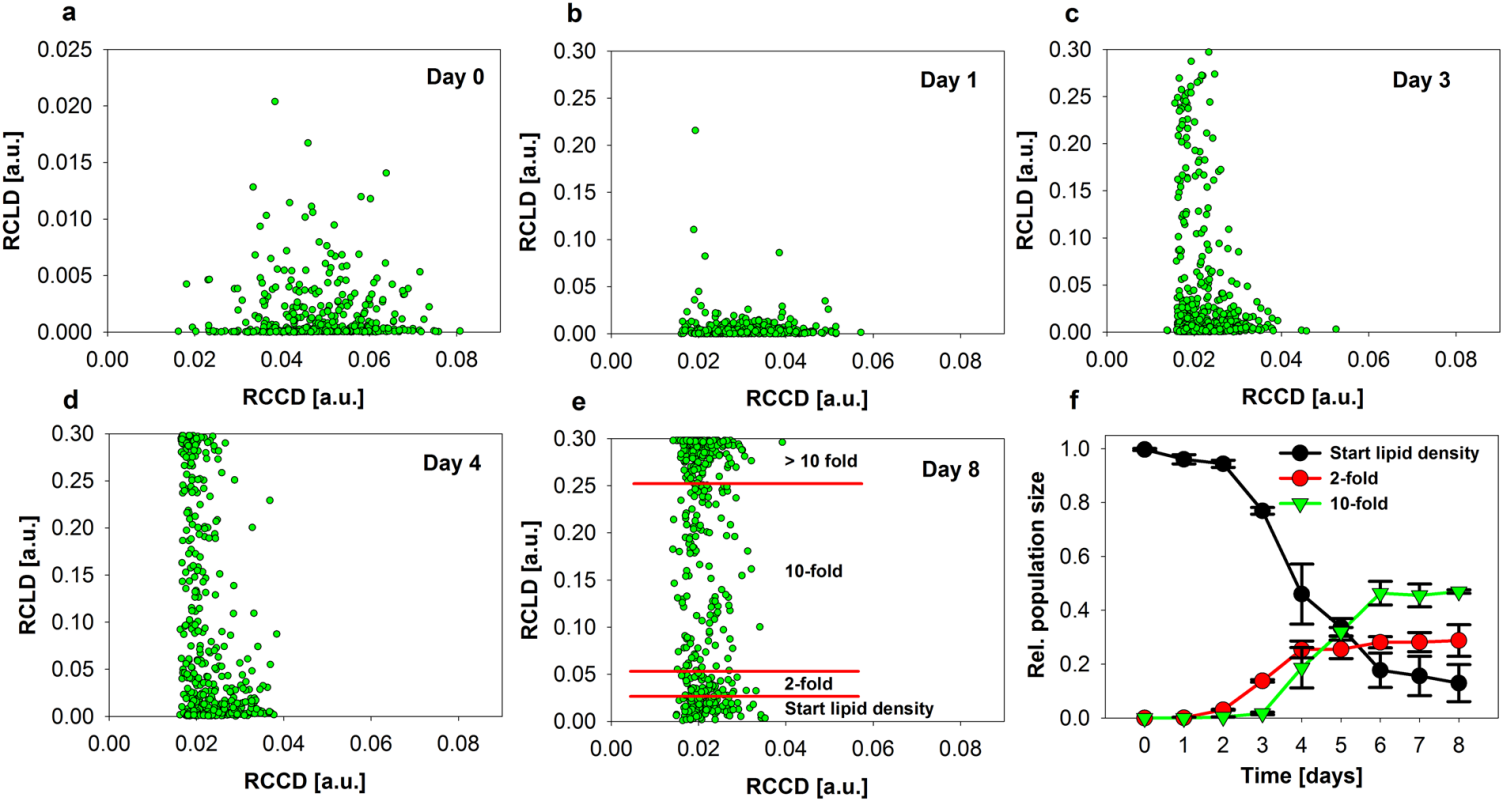
Scatter plots of the cellular chlorophyll and lipid density determined by single-cell analysis. Data from replicate 1 is shown in the scatter plots (**a** to **e**), and the visualization of the population size of transition states (**f**) contains mean values of both biological replicates ± S.D. Between 305 and 1012 cells are included in the particular datasets.

It was shown that strong changes in lipid and chlorophyll content occur during nitrogen starvation. Figure 4 displays two dimensional scatter plots between both measures on the level of single cells over time. A high cell-to-cell heterogeneity can be observed, and no clear dependency was found between both measures at day 0. Most of the cells exhibited a very low RCLD. After one day of nitrogen starvation, a few cells already started to increase their RCLD, while the culture became more heterogonous. In parallel, RCCD tended to decrease and the population became more homogenous (Supplementary Fig. S3). During the next days, the cells synthesized more and more TAG, while the RCCD still declined. Cells with lower RCCD tended to possess higher TAG contents (RCLD). After day four, the RCLD of some cells already reached the upper detection limit of the CCD-camera (values of 0.3). During the second half of the experiment, the cell culture started to split into two different subpopulations exhibiting very different RCLD. A more detailed analysis of this behavior was possible after defining so called transition states (red lines in Fig. 4e). All transitions states were related to the starting population at day zero. Figure 4f visualizes the trajectory of cells through the defined transition states. At the end of the experiment, around 10% of the cells still exhibited similar RCLD as at the beginning; 10% moved into the group with second-fold RCLD and around 50% of the cells exhibited more than a tenfold RCLD. From day four to day eight, relative size of the transition state “2-fold” and “10-fold” were constant over time indicating a parallel development of these particular cells. In the same time, the relative population size of transition state “Start-RCLD” decreased indicating that there is still a net accumulation of TAG until day eight.

### Multivariate data analysis for a comprehensive overview about the growth experiment

**Figure 5:**
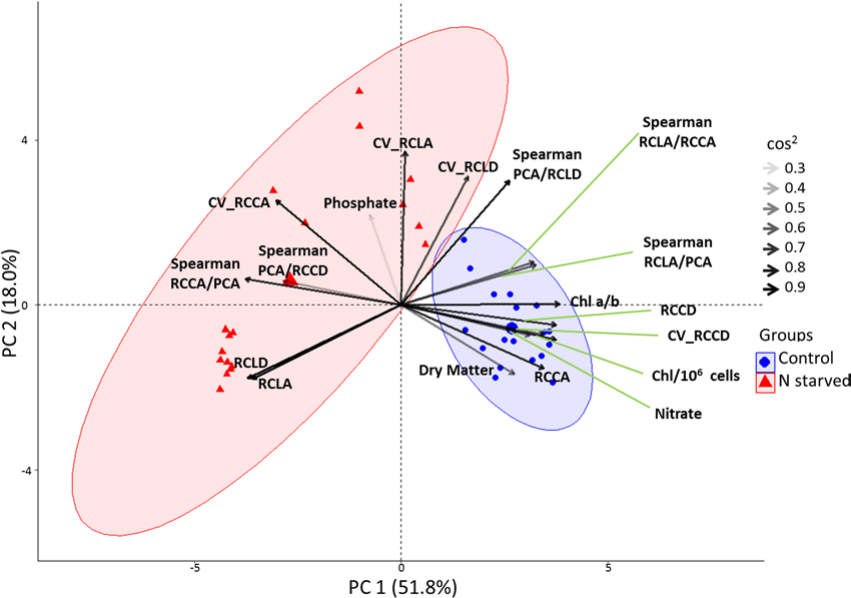
Principle component analysis of the variables and scores for the analyzed cultures (PCA - Biplot visualization).

A variety of bulk and single-cell parameters have been included in a multivariate data analysis (Fig. 5 and Supplementary Fig. S4). For example, total lipid content and fatty acid composition data from GC-FID analysis (Supplementary Table S3), mean cellular chlorophyll content (Fig. 2), TAG content derived from microscopy (Fig. 2), cell-to-cell heterogeneity (Supplementary Fig. S3), and correlation between different measures on the single-cellular level (Supplementary Tables S1 and 2). The Principle component analysis helps to visualize differences between the control and the -N-cultures within the combined dataset. The strong difference between both culture groups resulted in a clear separation of two different cluster clouds. Cos^2^ of the PCA analysis (gray shades in Fig. 5) describes the importance of the attribute to the given observation. Most of the parameters used have an intermediate or high influence, whereas for example the phosphate content of the cell media, dry matter content, and the Spearman rho between RCCA and PCA (RCS) have low influence in this dataset. Additionally, the result of a hierarchical cluster analysis of day eight of the experiment, including fatty acid composition, is shown in (Supplementary Fig. S4), where both culture groups deviate from each other within the majority of parameters.

## Discussion

Algal cells are very promising sources of lipids that that can be used as e.g., edible oils or as biofuels^36,37^. The green alga *Acutodesmus obliquus* is able to accumulate very high amounts of TAG, especially when cultivated under nitrogen starvation^1^. Yet, the complex dynamics during nitrogen starvation and formation of TAG containing LB is not satisfyingly understood. In this work, application of a novel multiparameter single-cell analytical technique was established and used in a biotechnological approach for stimulated lipid accumulation in cell cultures of *A. obliquus*. The microscopic cytometry based on epi-fluorescence microscopy, automated image analysis, and phenotypic characterization of the cells. General advantages of automated cell recognition algorithms are: (1) An impartial decision for object recognition and characterization based on strict rules^38^. (2) An image-analysis routine can be applied to a large number of microscopic images which enables the collection of large amounts of data for statistical analysis^38^. (3) A major advantage is the underlying intrinsic information about spatial organization and size of the objects. Based on cellular size information, concentrations of molecules can be determined within cells. In comparison to this, total cellular amounts of a molecules, as they are often measured by, e.g. FC, might be strongly affected by the respective cell size. This makes concentration (or density^23,24^) measures the more valuable information for theoretical considerations in, e.g. systems biology.

The presented technique is a simple but effective solution for unsupervised object recognition within microscopic images, characterisation of the identified objects, and an integrated data handling routine for quantitative single-cell analysis which means characterisation of population structures, and data visualisation. The key of the unsupervised object recognition routine is its simplicity. In this routine, a combination between use of the intrinsic chlorophyll fluorescence of algal cells as a trigger signal, to identify the chloroplasts/objects, and the use of a post processing contrast enhancement method to determine the cell body surrounding the chloroplast. Based on this step the pixels belonging to distinct cells can be identified without knowledge of cellular geometry or uniformity of cells. A scheme of this procedure is given in Supplemental Fig. S1. As signals, intrinsic chlorophyll fluorescence and the well-investigated fluorescent dye NR was used to stain neutral lipids inside the cells. After cell recognition, chlorophyll amount, lipid amount, and the corresponding compound densities were quantified on a single-cellular level. It has to be noted, that parallel determination of various cellular ingredients by fluorescence analysis (e.g. FC or quantitative microscopy) could be disturbed by several optical effects, like energy transfer from one chromophore species to another. The impact of these effects is highly dependent on the setup used for the measurements and cannot be generalized. By comparison with results from traditional reference analysis, it was shown that this technique is able to follow time-dependent development of chlorophyll of non-stressed and nitrogen starved cultures and showed a good correlation with results from spectroscopic quantification of cellular NR fluorescence (Supplementary Fig. S2). Based on the presented data, contribution of potentially disturbing optical effects, within the established microscopic method, was shown to be minor.

During the experiment, cells exhibited a very high cell-to-cell heterogeneity in each of the particular parameters (Figs 3 and 4 and Supplementary Fig. S3). Furthermore, cells showed a high correlation between cell size and chlorophyll amount, but a very low and dynamic correlation between cell size and lipid amount and even a slight negative correlation between cell size and lipid density (lipid amount normalized with regard to cell size) (Fig. 3 and Supplementary Tables S1 and 2). Similar results were described in studies about single-cell dynamics of starch in synchronous algal cultures^23–25^. There, the cells possessed a high cell-to-cell heterogeneity and the relation between *relative cellular starch density* (RCSD) and cellular size, which were very similar as RCLD evaluated in the present study (Fig. 3d). In the preceding work, small cells tended to contain higher RCSD with very low correlation coefficients between RCSD and cell size. Including data evaluated in the present work, this situation was described for different algal species and strains under very different growth conditions and could display a more general relationship for storage compounds in green algae. After induction of nitrogen starvation at day zero of the present study, the cultures characteristically changed their mean chlorophyll and lipid content (Figs 1 and 2). The microscopic single-cell analytical technology was able to follow changes of cell-to-cell heterogeneity of the particular parameters over time (Supplementary Fig. 3). The RCLD heterogeneity showed a strong and abrupt increase, even before the mean lipid content significantly increased. The cells responded very fast to the nitrogen starvation and a small fraction of cells increased their lipid content dramatically already after one day of starvation and cells with lower RCCD tended to possess higher TAG contents.

After several days, the culture differentiated into two subpopulations with very different RCLD. The time-dependent analysis of the cellular transition within the subpopulations indicated that the cells still accumulate TAG and did not fall back from high RCLD to low RCLD (Fig. 4f). Jaeger *et al*. and Cavonius *et al*. recently investigated intracellular lipid droplets in other algal cells with complex microscopic setups including multiple lasers and time-gated detectors^26,27^. In their study, CARS signals were used for label-free microscopy of TAG with the aim to exclude the chlorophyll fluorescence which disturbs the faint Raman signals of the fatty acids. The highly advanced microscopic imaging approaches mentioned in this work, including SHG microscopy, are clearly breakthrough technologies, but each technique has its own advantages and disadvantages. Disadvantages are e.g., complex optical setups and the high investment costs. In this work a simple, fast, and cheap procedure was used to study single-cell dynamics of algal cells during nitrogen starvation. This basic setup was able to evaluate 25 dimensions (measured and derived) that describe the population structure over time. Some of the parameters react very sensitive on changes of culture conditions induced. Within most of the parameters, the starved culture differed clearly from the control culture. This is also illustrated in the PCA and cluster analysis of the datasets (Fig. 5 and Supplementary Fig. S4). In principle, a fully automated sample preparation and direct coupling of microscopes to bioreactors is feasible by microfluidics and video microscopy^12^. Such multi-dimensional single-cell analysis can be used for an advanced process monitoring and control during biotechnological production, because the single-cell datasets obtained are a very sensitive tool to follow trajectories of cellular development during a production process. Due to a higher time resolution based on automatization, the complex interplay of cellular physiology with changing environmental conditions in biotechnological processes might be understood on a higher level.

Reasons for the cell-to-cell heterogeneity at the different time points and especially, the dynamic split into two strongly different subpopulations can be potentially stochasticity in gene expression^39^, metabolic activity^39^, because of asymmetric cell division^24^, asymmetric partitioning of storage compounds^24^, or of central signal molecules with low abundance upon cell division^40^. Additionally, external factors such as external perturbations or fluctuations in the cellular surroundings can cause cell-to-cell heterogeneity^40^. In the end, a combination of the different phenomena is realistic and even more complex physiological circumstances should be considered. It might be that the physiological reaction of the cells to cope with the nitrogen starvation could be delayed in a significant number of cells that are still busy with other critical processes like cell division and daughter cell release. If there are such critical overriding processes, a cell population could be splitted into different populations with completely different properties. However, major questions remain for all biotechnological processes. 1) How can productivity be influenced to obtain higher product yields? 2) After having experimental access to the complex population structures, the question raises how the biochemical composition of the product quality e.g. fatty acid composition is distributed across the cell populations. These questions have to be answered in the future. With each development on the level of microscopic setups and of novel object recognition algorithms, single-cell analytical technologies will be pushed forward. This enables novel insights, for systems biology and bioprocess monitoring, of how cell populations differentiate based on changing environmental conditions or after external stimuli.

## Materials and Methods

See Supplementary Methods for additional information.

### Cell culture conditions

*Acutodesmus obliquus (*SAG 276-10) was cultured in lab-scale bubble columns (1.8 L suspension volume each) under continuous light (130 µmol photons m^−^² s^−1^) at 25 °C and 3% CO_2_ [v/v] in a synthetic growth media called ½ Tamiya^41^. Two independent biological replicates of the control and the nitrogen starved cultures were cultivated in parallel, respectively. Inoculum cultures were derived from two batches grown under identical conditions. Isogenic cultures were obtained by recloning (see Supplementary Methods for details). Nitrogen starvation was induced shortly before start of the experiment through exchange of the growth media (centrifugation at 3,000 × g for 10 min) with nitrogen lacking media. Control cultures have been treated identical but suspended in the full growth media. Measured nitrate contents are shown in Supplementary Fig. S5.

### Neutral lipid staining

The basic staining procedure with nile red (NR) was based on information from^29,30^. See Supplementary Methods for details. NR-dependent fluorescence was quantified by either fluorescence spectroscopy or fluorescence microscopy.

### Fluorescence microscopy

Fluorescence microscopy was done with a DM5500B microscope (Leica Microsystems GmbH, Wetzlar), equipped with a 40 × objective (Leica 40 × HCX PL Fluotar, NA 0.75). Nile red was excited at 546 nm (BP 546/12) and the fluorescence was detected after passing a band-pass filter (BP 600/40). Excitation of the chlorophyll fluorescence was done at 620 nm (BP 620/60) Separation of fluorescent light emitted by the chlorophyll of the cells was done by a band-pass filter (BP 700/75).

### Image analysis

Raw images were processed using a custom-designed routine in MATLAB (The MathWorks, Natick, USA). Object recognition steps and technical details have been described elsewhere^19^. The established routine was extended to include cell size determination, chlorophyll amount and content and lipid amount and content as primary signals followed by distribution characterization and correlation analysis steps. Briefly: the routine based on the following steps: Contrast enhancement of the microscopic image from the chlorophyll fluorescence data, conversion of RGB data to grayscale, automated reduction of the gray level image to a binary image was done according to^28^ which includes the discrimination between background and objects, removal of very small objects from the image, and assignment of pixels to distinct cells. After this, number of pixels belonging to the cell and equivalent sphere diameter can be determined. Analysis of single-cell chlorophyll and lipid was done as follows: re-loading of the unmodified fluorescence image for chlorophyll fluorescence, converting of RGB image to grayscale, cross checking of pixel coordinates from object recognition routine, readout of the distinct coordinates in the unmodified image, and summing up fluorescence intensities of the distinct cells. This loop was additionally repeated with the lipid raw data (NR fluorescence data). Additional information about data generation, interpretation, and particle sizing is given in the Supplementary Methods.

### Determination of the fatty acid profile

The lipid extraction based on methods according Folch *et al*. (1957) and Bligh and Dyer with slight modifications^8,9^. The fatty acid profiles of the algae were determined as fatty acid methyl esters (FAME).

### Multivariate data analysis

The principal component analysis was performed with R (Version 3.4.1., R Development Core Team (2008). R: A language and environment for statistical computing (R Foundation for Statistical Computing, Vienna, Austria).

### Data availability

The datasets generated during and/or analyzed during the current study are available from the corresponding author on reasonable request.

## Electronic supplementary material

Supplementary Dataset 1

## References

[1] de Jaeger, L. *et al*. Superior triacylglycerol (TAG) accumulation in starchless mutants of *Scenedesmus obliquus*: (I) mutant generation and characterization. *Biotechnology for Biofuels* **7**, 10.1186/1754-6834-7-69 (2014). 10.1186/1754-6834-7-69 PMC4052810 24920957

[2] Hu Q (2008). Microalgal triacylglycerols as feedstocks for biofuel production: perspectives and advances. Plant J..

[3] Breuer G (2013). Effect of light intensity, pH, and temperature on triacylglycerol (TAG) accumulation induced by nitrogen starvation in *Scenedesmus obliquus*. Bioresour Technol..

[4] Santos AM (2012). Growth of oil accumulating microalga *Neochloris oleoabundans* under alkaline-saline conditions. Bioresour Technol.

[5] Ledford HK, Niyogi KK (2005). Singlet oxygen and photo-oxidative stress management in plants and algae. Plant Cell Environ.

[6] Zhang, Y-M., Chen, H., He, C-L., & Wang, Q. Nitrogen Starvation Induced Oxidative Stress in an Oil-Producing Green Alga *Chlorella sorokiniana* C3. Appanna VD, ed. *PLoS ONE*. 8:e69225, 10.1371/journal.pone.0069225 (2013). 10.1371/journal.pone.0069225 PMC3712941 23874918

[7] Goncalves EC, Wilkie AC, Kirst M, Rathinasabapathi B (2016). Metabolic regulation of triacylglycerol accumulation in the green algae: identification of potential targets for engineering to improve oil yield. Plant Biotechnology Journal..

[8] Folch J, Lees M, Stanley GHS (1957). A simple method for the isolation and purification of total lipides from animal tissues. Journal of Biological Chemistry..

[9] Bligh EG, Dyer WJ (1959). A rapid method of total lipid extraction and purification. Canadian Journal of Biochemistry and Physiology..

[10] Amantonico A, Urban PL, Zenobi R (2010). Analytical techniques for single-cell metabolomics: state of the art and trends. Anal Bioanal Chem..

[11] Wang D, Bodovitz S (2010). Single cell analysis: the new frontier in ‘omics’. Trends Biotechnol..

[12] Fritzsch FSO, Dusny C, Frick O, Schmid A (2012). Single-Cell Analysis in Biotechnology, Systems Biology, and Biocatalysis. Annual Review of Chemical and Biomolecular Engineering.

[13] Glassey J (2011). Process analytical technology (PAT) for biopharmaceuticals. Biotechnol. J..

[14] Broger T, Odermatt RP, Huber P, Sonnleitner B (2011). Real-time on-line flow cytometry for bioprocess monitoring. J. Biotechnol..

[15] Lencastre FR (2011). Experimental methods and modeling techniques for description of cell population heterogeneity. Biotechnol. Adv..

[16] Enfors SO (2001). Physiological responses to mixing in largescale bioreactors. J. Biotechnol..

[17] Shapiro H. M. *Practical flow cytometry* (John Wiley and Sons, 2003).

[18] Hyka P, Lickova S, Přibyl P, Melzoch K, Kovar K (2013). Flow cytometry for the development of biotechnological processes with microalgae. Biotechnology Advances.

[19] Sandmann M, Lippold M, Saalfrank F, Odika CP, Rohn S (2017). Multi-dimensional single-cell analysis based on fluorescence microscopy and automated image analysis. Anal. Bioanal. Chem..

[20] Elfwing A, LeMarc Y, Baranyi J, Ballagi A (2004). Observing growth and division of large numbers of individual bacteria by image analysis. Appl. Environ. Microbiol..

[21] Matsumura K, Yagi T, Yasuda K (2003). Role of timer and sizer in regulation of Chlamydomonas cell cycle. Biochem. Biophys. Res. Commun..

[22] Schönholzer F, Hahn D, Zarda B, Zeyer J (2002). Automated image analysis and *in situ* hybridization as tools to study bacterial populations in food resources, gut and cast of *Lubricus terrestris L*. J. Microbiol. Methods.

[23] Garz A (2012). Cell-to-cell diversity in a synchronized Chlamydomonas culture as revealed by single-cell analysis. Biophys. J..

[24] Rading, M. *et al*. Weak correlation of starch and volume in synchronized photosynthetic cells. *Phys Rev E*. **91**. 10.1103/PhysRevE.91.012711 (2015). 10.1103/PhysRevE.91.012711 25679646

[25] Sandmann M, Garz A, Menzel R (2016). Physiological response of two different Chlamydomonas reinhardtii strains to light-dark rhythms. Botany..

[26] Jaeger D (2016). Label-free *in vivo* analysis of intracellular lipid droplets in the oleaginous microalga *Monoraphidium neglectum* by coherent Raman scattering microscopy. Scientific Reports..

[27] Cavonius, L. *et al*. Imaging of lipids in microalgae with CARS-microscopy. *Plant Physiology*, 10.1104/pp.114.252197 (2015). 10.1104/pp.114.252197 PMC4348760 25583924

[28] Otsu N (1979). A threshold selection method from gray-level histograms. IEEE Trans Syst Man Cybern..

[29] Rumin, J. *et al*. The use of fluorescent Nile red and BODIPY for lipid measurement in microalgae. Biotechnology for Biofuels. **8**, 10.1186/s13068-015-0220-4 (2015). 10.1186/s13068-015-0220-4 PMC4364489 25788982

[30] Chen W, Sommerfeld M, Hu Q (2011). Microwave-assisted Nile red method for *in vivo* quantification of neutral lipids in microalgae. Bioresource Technology..

[31] da Silva TL, Reis A, Medeiros R, Oliveira AC, Gouveia L (2009). Oil Production Towards Biofuel from Autotrophic Microalgae Semicontinuous Cultivations Monitorized by Flow Cytometry. Appl. Biochem. Biotechnol..

[32] de la Jara A (2003). Flow cytometric determination of lipid content in a marine dinoflagellate, Crypthecodinium cohnii. Journal of Applied Phycology.

[33] Greenspan P, Mayer EP, Fowler SD (1985). Nile red: a selective fluorescent stain for intracellular lipid droplets. J Cell Biol..

[34] Gusbeth, C. A. *et al*. Fluorescence Diagnostics for Lipid Status Monitoring of Microalgae during Cultivation. International Journal of Renewable Energy and Biofuels, **2016**, 10.5171/2016.899698r (2016).

[35] Laurens LML, Wolfrum EJ (2011). Feasibility of Spectroscopic Characterization of Algal Lipids: Chemometric Correlation of NIR and FTIR Spectra with Exogenous Lipids in Algal Biomass Bioenerg. Res..

[36] Breuer G (2012). The impact of nitrogen starvation on the dynamics of triacylglycerol accumulation in nine microalgae strains. Bioresour Technol..

[37] Griffiths MJ, van Hille RP, Harrison EH (2012). Lipid productivity, settling potential and fatty acid profile of 11 microalgal species grown under nitrogen replete and limited conditions. Journal of Applied Phycology..

[38] Arce SH, Wu P-H, Tseng Y (2013). Fast and accurate automated cell boundary determination for fluorescence microscopy. Sci Rep..

[39] Delvigne F, Zune Q, Lara AR, Al-Soud W, Sørensen SJ (2014). Metabolic variability in bioprocessing: implications of microbial phenotypic heterogeneity. Trends in Biotechnology..

[40] Rosenthal, K., Oehling, V., Dusny, C., Schmid, A. Beyond the bulk: disclosing the life of single microbial cells, FEMS Microbiology Reviews, fux044 (2017). 10.1093/femsre/fux044 PMC5812503 29029257

[41] Hase E, Morimura Y, Tamiya H (1957). Some data on the growth physiology of Chlorella studied by the technique of synchronous culture. Arch Biochem Biophys..

