# Supplementary material for: Analysis of population structures of the microalga Acutodesmus obliquus during lipid production using multi-dimensional single-cell analysis
Source: Sci Rep. 2018 Apr 19;8:6242. doi: 10.1038/s41598-018-24638-y (PMC5908859; doi:10.1038/s41598-018-24638-y)
Supplement: Supplementary file 1 — Supplementary Dataset 1 [file 41598_2018_24638_MOESM1_ESM.docx]

**Analysis of population structures of the microalga *Acutodesmus obliquus* during lipid production using multi-dimensional single-cell analysis**

Michael Sandmann^1*^, Michaela Schafberg^2^, Martin Lippold^1^, and Sascha Rohn^1,2^

*^1^Institute for Food and Environmental Research (ILU e. V.), Arthur-Scheunert-Allee 40-41, 14558 Nuthetal, Germany*

*^2^Hamburg School of Food Science, Institute of Food Chemistry, University Hamburg, Grindelallee 117, 20146 Hamburg, Germany*

**Corresponding author:**

Michael Sandmann

[michael.sandmann@ilu-ev.de](mailto:michael.sandmann@ilu-ev.de)

+4933200518815

**Supplemental Methods**

**Biological material.** The Strain was obtained from the SAG Culture Collection of Algae (Göttingen, Germany)

**Cell culture media compoosition.** The final concentration of the macro nutrients is: KNO_3_ 2.5 g/L, KH_2_PO_4_ 0.625 g/L, MgSO_4_*7H_2_O 1.25 g/L, FeSO_4_*7H_2_O 4.5 mg/L and Na_2_EDTA 18.6 mg/L. The final concentration of the minor components is: H_3_BO_3_ 1.43 mg/L, MnCl_2_*4H_2_O 0.905 mg/L, ZnSO4*7H2O 0.111 mg/L, MoO_3_ 9 µg/L, and NH_4_VO_3_ 11.5 µg/L. Both pre-culture and the growth experiments were performed under axenic conditions.

**Culture handling.** Recloning was based on standard procedures using sterile technique for isolation of algal cells from natural habitats^1^. Briefly, strong dilutions (1:10,000) of algal cell suspension with fresh culture media have to be prepared following spread of small aliquots of the diluted suspension on agar plates containing cell culture media, growth of cells to colonies and picking of single colonies to start a new propagation cycle. The whole procedure has to be repeated several times to obtain an isogenic culture^1^.

Samples were taken each day at 10 AM with a sterile 20 mL syringe from the sample port. The sample port was integrated in the head of the bubble column and was cleaned freshly with ethanol before each sample taking procedure. Positive pressure through the gas supply and a negative pressure inside the coupled syringe ensures no backflow of suspension when gently opening the sample port valve. 20 mL of cell suspension were discarded first, and the sample was taken afterwards.

**Quantification of dry matter content.** Dry matter content of the suspension was determined gravimetrically^2^.

**Pigment extraction and quantification.** Aliquots of the cell suspension (2 mL suspension each) were collected by centrifugation (5 min at 3,000 × g), frozen, and stored at -20°C. For pigment extraction, cells were resuspended in 5 mL 80% [v/v] acetone and 1 g of glass beads (diameter 0.25 to 0.5 mm) was added to the suspension. Cells were broken in a bead mill (Retsch MM 301, Retsch, Haan, Germany) with (30 Hz shaking frequency, 15 min duration). Following the breakage procedure, cells were centrifuged (5 min at 3,000 × g) and the supernatants used for pigment analysis. Chlorophyll a, chlorophyll b and the total carotenoid content of the extracts were determined photometrically at 470, 647, and 663 nm, respectively^3^.

**Quantification of cell number and cell size.** Cell number and cell size were determined using a ‘MULTISIZER 3’ device according the instructions of the manufacturer (Beckman Coulter, Krefeld, Germany).

**Neutral lipid staining.** The staining procedure with nile red (NR) was based on information from^4,5^. Cell number was adjusted to 10 million cells per mL with fresh growth media and the cells were incubated for 5 min at 37 °C with DMSO (1 % [v/v]) in a thermo shaker at 1000 rpm. Subsequently, nile red (NR; Sigma, Deisenhofen, Germany) dissolved in acetone was added to the cell suspension (final concentration 2 µg/mL). The final mixture was incubated for 20 min at 37 °C in a thermo shaker at 1,000 rpm. NR treated cells were kept in dark before analysis. NR-dependent fluorescence was quantified by either fluorescence spectroscopy or fluorescence microscopy. In case of controls without NR, acetone without NR was used. The rest of the treatment was identical to NR stained cells.

**Image analysis.** Spectral information from the different field of views in each sample was stored in a stack of images with image size of 304 × 228 µm (1600 × 1200 pixels). Raw images were processed using a custom-designed routine in MATLAB (The MathWorks, Natick, USA). Object recognition steps and technical details have been described elsewhere^1^. A major advantage of microscopic cytometry is the intrinsically given size information of the objects. Within the optical path of the epi-fluorescent microscope, 3-dimensional information of a single cell is converted into a projected 2-dimensional information. Diameter or volume of objects can be calculated from this projected information assuming spheroidal shape which results in the commonly used equivalent sphere diameter for particle characterization. Cells with a flat cell morphology, like *Acutodesmus obliquus*, exhibit significant overestimation of the calculated equivalent sphere diameter of e.g., by a factor of two^2^. A flat cell morphology is also a challenge for other particle sizing technologies, like the well-known Coulter counter^1^. For such flat cell morphology, the directly obtained projected cell area was used as a meaningful size measure for cells. The size of the PCA is represented by the number of pixels or by an absolute surface area. A single pixel represents the surface of 0.0361 µm². In this work, PCA is further on labeled with *relative cell size* (RCS) and the more convenient pixel number was used for quantification of the RCS. Based on emitted fluorescence from the chlorophyll or from the fluorescent dye NR, relative chlorophyll amounts of each distinct cell and relative neutral lipid amounts can be quantified. The cellular constituents quantified by the image analysis are given either as relative constituent amount (sum of the detected fluorescent light by the CCD-camera over all pixels belonging to a distinct cell) or as relative constituent density (given as relative constituent amount divided by the number of pixels from the RCS of the corresponding cell). The first one corresponds for a constituent amount per cell and the latter one for a constituent concentration per cell. A so-called “compensation method” for the optical phenomena, as it is sometimes used in flow cytometry^6^, was not applied in this study.

**Determination of the fatty acid profile.** The algae cultures were lyophilized and 10-15 mg algae dry mass was mixed with 2 mL Ethanol, 3.95 mL *t*BME and 0.05 mL heptadecanoic acid (2 mg mL^-1^ in *t*BME). The solution was sonicated for 15 min at room temperature and then shaken for 10 min. This extraction was repeated once. 7.5-11 mg activated charcoal was added, shaken for 5 min and centrifuged at 3,226 *x* g for 15 min at 4 °C. 1.5 mL of the supernatant were dried under nitrogen stream and re-suspended with 50 µL *t*BME. For the determination as fatty acid methyl esters the extract was derivatized with 25 µL TMSH and injected to GC or stored at -20 °C until GC analysis. The fatty acid composition was determined by GC-FID on a CP-SIL 88 column (50 m x 0.25 mm x 0.20 µm) installed on an Agilent 6890 gas chromatograph (Agilent Technologies Inc., Santa Clara, USA) equipped with FID, an Agilent 550 controller, Agilent autosampler 7683 and a PC. The initial oven temperature was 150 °C, held for 5 min, increased to 230 °C at a rate of 15°C min^-1^ and held for 30 min. The carrier gas was Nitrogen with a flow rate of 1.8 mL min^-1^ with constant pressure of 360 kPa. The injector was set at 240 °C and the detector was set at 260 °C. The split was 75 mL min^-1^ and the split ratio was 42:1. Fatty acids, respectively the FAME were identified by comparing their retention times with the fatty acid methyl ester standard mix containing 37 of the most common fatty acid methyl esters (‘FAME 37’). Each fatty acid was expressed as % of total lipid content.

**Multivariate data analysis.** The principal component analysis was performed with R (Version 3.4.1., R Development Core Team (2008). R: A language and environment for statistical computing. R Foundation for Statistical Computing, Vienna, Austria. ISBN 3-900051-07-0, URL http://www.R-project.org.) and R Studio (Version 1.0.136; RStudio Team (2016). RStudio: Integrated Development for R. RStudio, Inc., Boston, MA URL http://www.rstudio.com/) by using the following packages ade4 [1.7-5], ggplot2 [2.2.1] and factoextra [1.0.4]. For the representation of the heat map the packages d3heatmap [0.6.1.1] was used.

**Fluorescence spectroscopy.** Spectroscopic analysis of the NR stained samples was done with a FluoroMax4 (Horiba, Berlin, Germany). Excitation of the fluorescence was done at 488 nm. Total emission was quantified between 500 nm and 900 nm with 1 nm resolution.

**Supplemental Results**

**
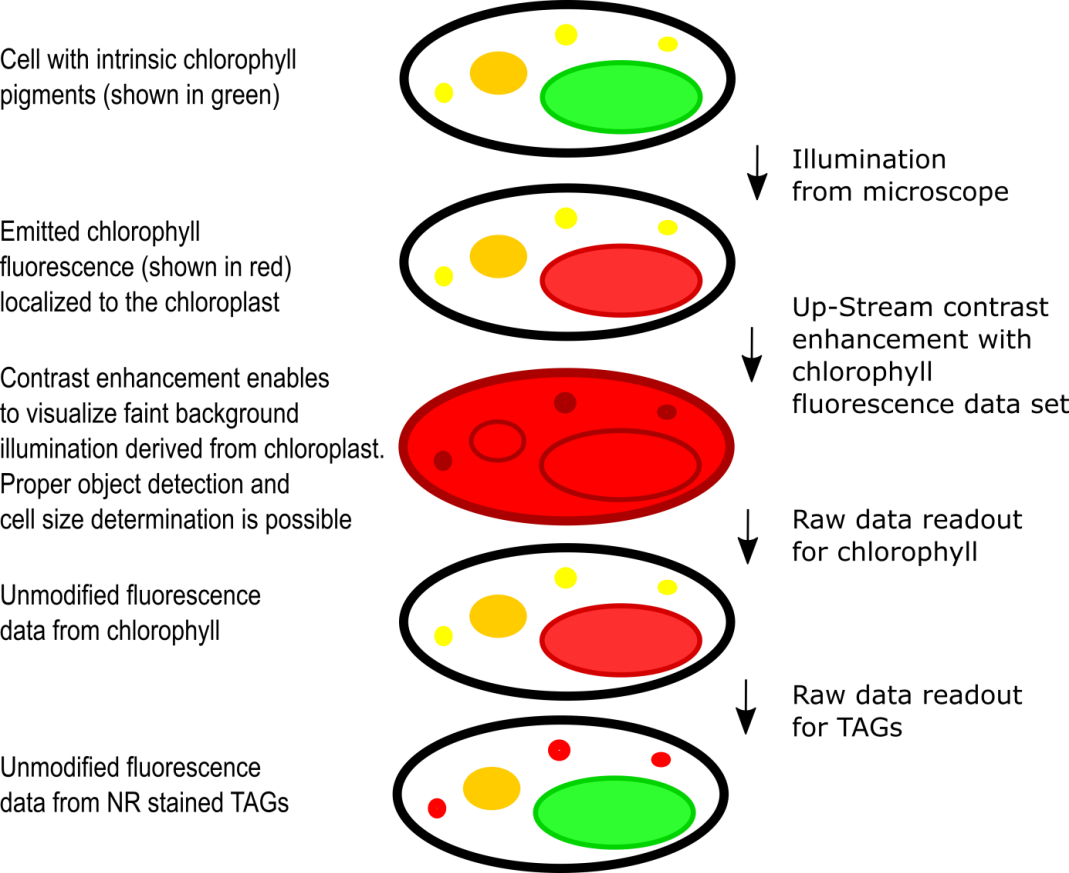
**

**Figure S1:** **Schematic drawing of the relationship between primary fluorescence emission, illumination of the rest of the cell, and analysis of cellular size, chlorophyll amount and lipid amount.** (Please note this procedure was established for the green alga *A. obliquus*. It should be applicable in numerous cell cultures, but validity of the object recognition routine should be proven by reference methods.)


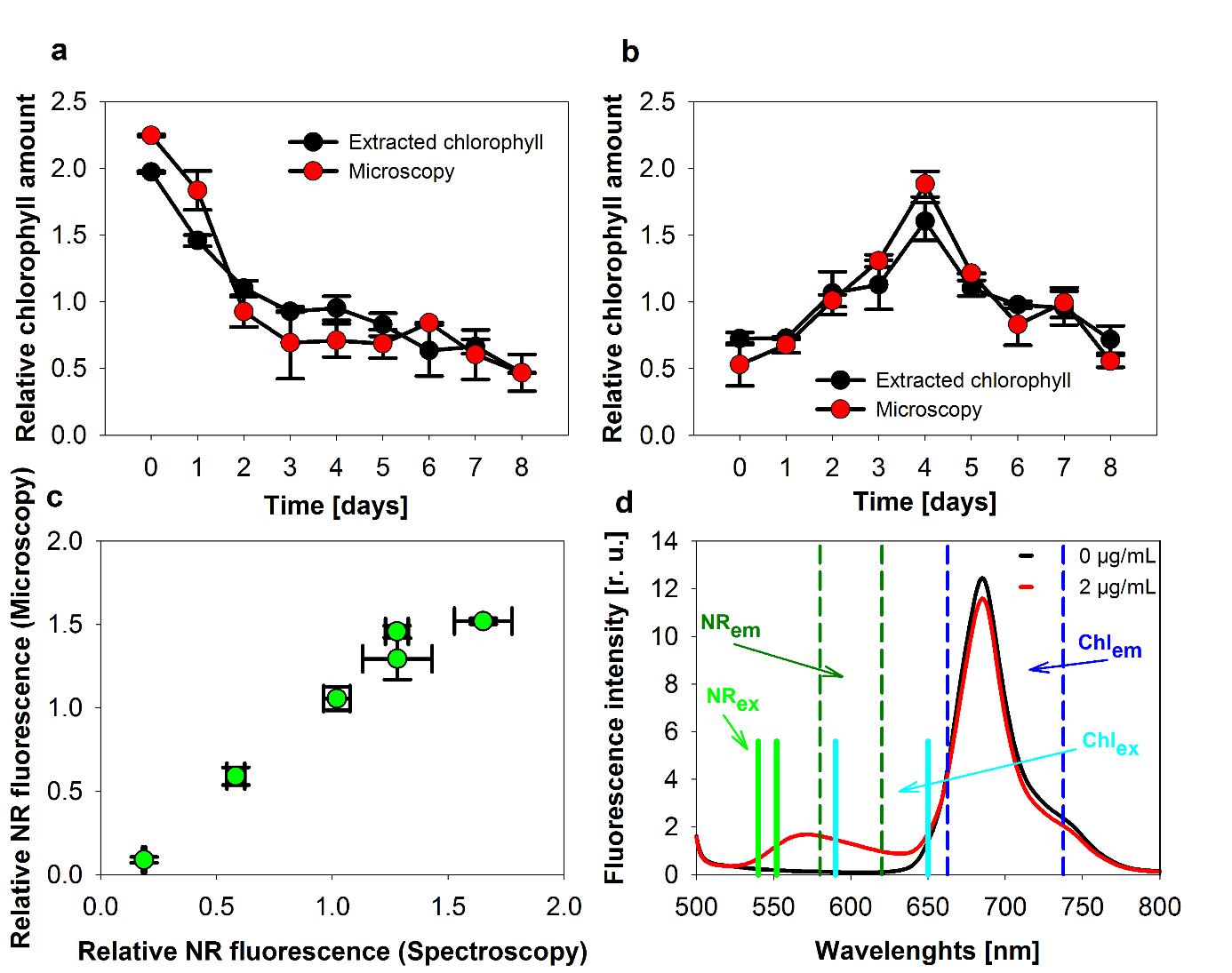


**Figure S2. Reliability of the imaging based single-cell approach.** For (a to c), data from different techniques was normalized with respect to the overall mean of the dataset to enable a direct comparison between both techniques on the same scale. (a) Chlorophyll amount per cell under nitrogen starvation (b) Chlorophyll amount per cell under control conditions. For (a) and (b) each value is the mean of two biological replicates ± SD. Untransformed reference data for chlorophyll dynamics was shown before in Fig. 2b. (c) Microscopic and fluorescence quantification by spectroscopy with 6 different NR concentrations. Mean of technical replicates ± SD is given (n = 2 for microscopy and n = 3 for spectroscopy). (d) Representative fluorescence emission spectra from non-stained (black line) and NR-stained (red line) cells of *A. obliquus* are shown. Excitation wavelength was 488 nm. Vertical lines represent the spectral ranges of the used bandpass filter sets used in the microscope (see methods section for details). Spectral ranges for NR excitation (NR_ex_) and NR emission (NR_em_) are shown in green. Spectral ranges for chlorophyll excitation (Chl_ex_) and chlorophyll emission (Chl_em_) are shown in blue.


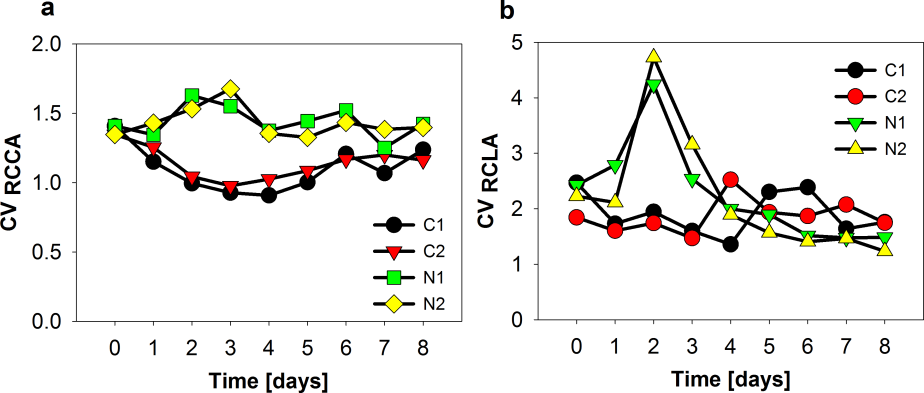


**Figure S3. Single-cell data sets of relative chlorophyll and lipid content.** Changes of coefficient of variation (CV) of relative chlorophyll (a) and relative lipid (b) distributions over time. Between 305 and 1012 cells are included in the datasets. The coefficient of variation (CV) is a relative measure of the width of a distribution and can be used to describe cell-to-cell dynamics quantitatively over time^7,8^. Changes of CV can be interpreted as nonparallel developments of the cells in suspension resulting ether in an increased or diminished heterogeneity. The observed heterogeneity was very high in all cultures. Dynamics of the C.V. point to changes in the cell-to-cell heterogeneity and can be used to reveal concrete time points in which the structure of distinct cell population changes.

**
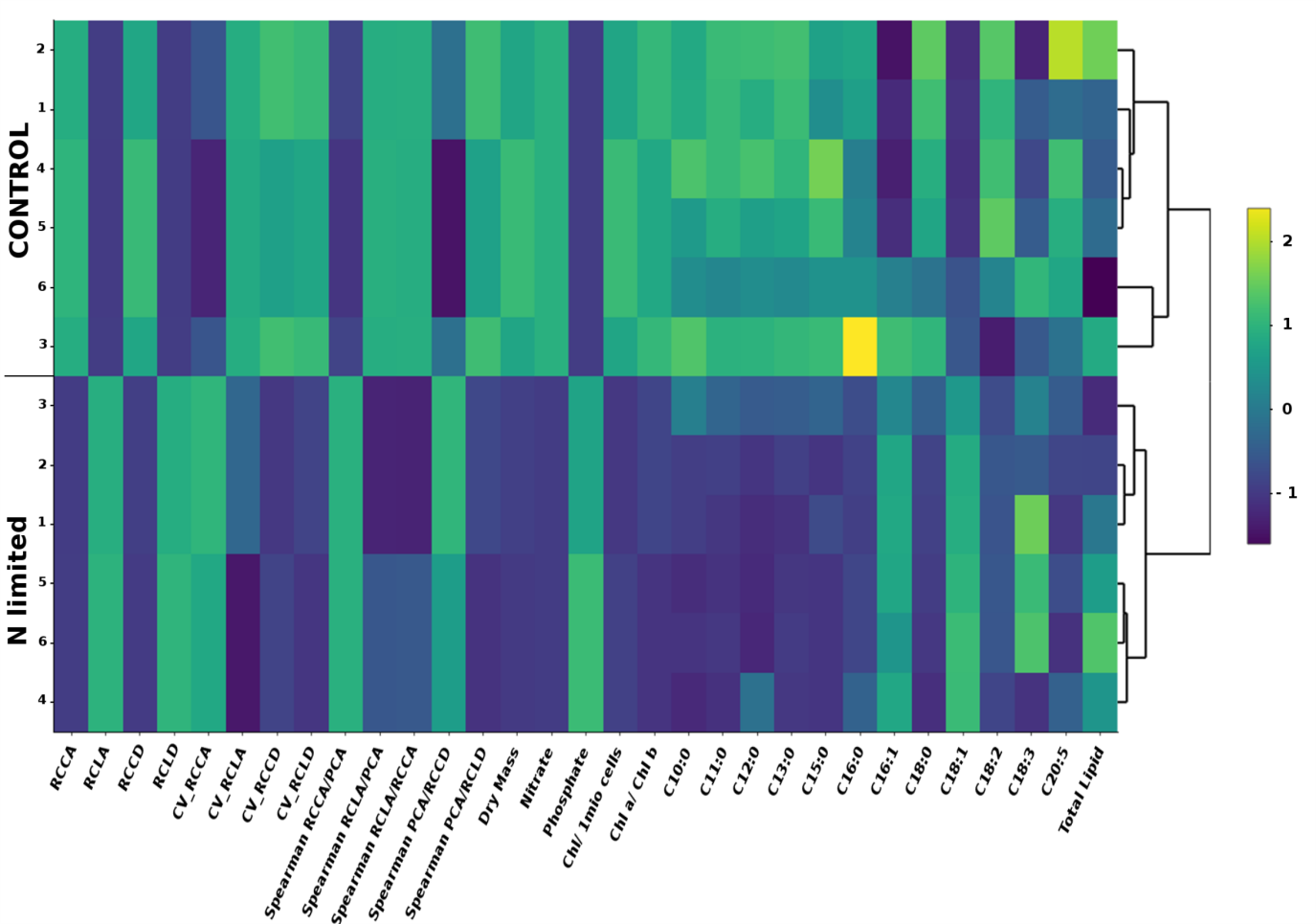
**

**Figure S4. Representation of the cluster analysis from various measured parameters characterizing control and –N-culture.** Heat map illustration shows the hierarchical clustering of the different cultivated groups. The graphic illustrates the contrasting behavior of the cultures blue colors indicate low values, whereas yellow colors indicate higher values. Technical replicates are shown for
–N-conditions and control conditions. Most parameters in both groups are contrary pronounced.


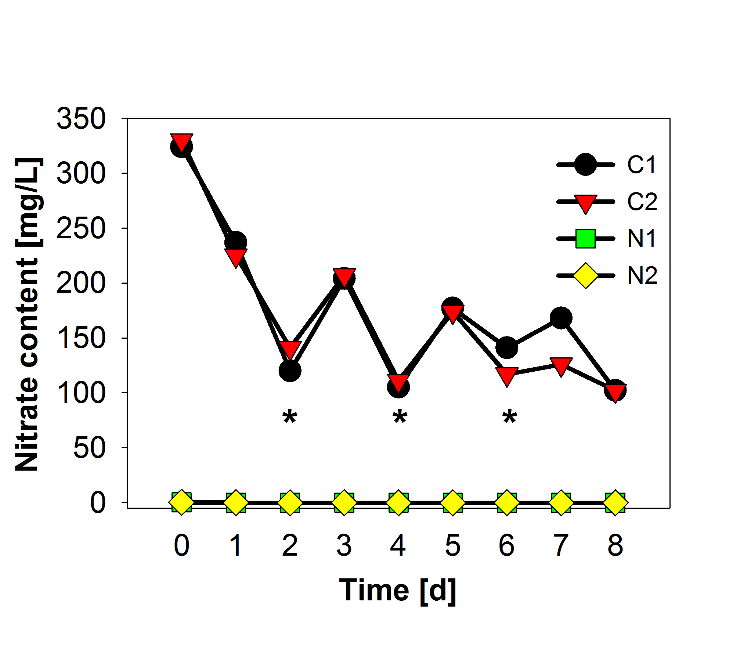


**Figure S5. Development of nitrate in starved and control cultures of *Acutodesmus o*.** Asterisks indicate time points for addition of nitrate for the control cultures, to avoid limitation. Single measurements are shown.

**Table S1:** Spearman rank correlation coefficients from replicate 1.

|  | **Spearman Rho for relative amount of chlorophyll against RCS** | | **Spearman Rho for relative density of chlorophyll against RCS** | | **Spearman Rho for relative amount of TAG against RCS** | | **Spearman Rho for relative density of TAG against RCS** | |
| --- | --- | --- | --- | --- | --- | --- | --- | --- |
| **Time [days]** | **C1** | **-N1** | **C1** | **-N1** | **C1** | **-N1** | **C1** | **-N1** |
| 0 | 0.955 | 0.960 | 0.209 | 0.115 | 0.579 | 0.594 | 0.177 | 0.329 |
| 1 | 0.952 | 0.982 | -0.106 | 0.372 | 0.780 | 0.775 | 0.228 | 0.404 |
| 2 | 0.974 | 0.988 | 0.317 | 0.390 | 0.687 | 0.705 | -0.030 | 0.320 |
| 3 | 0.967 | 0.992 | 0.217 | 0.512 | 0.716 | 0.572 | -0.046 | 0.104 |
| 4 | 0.961 | 0.991 | 0.313 | 0.450 | 0.802 | 0.231 | 0.133 | -0.250 |
| 5 | 0.958 | 0.990 | 0.406 | 0.402 | 0.665 | 0.395 | 0.060 | -0.214 |
| 6 | 0.965 | 0.987 | 0.406 | 0.377 | 0.591 | 0.484 | -0.023 | -0.230 |
| 7 | 0.965 | 0.990 | 0.419 | 0.408 | 0.817 | 0.447 | 0.185 | -0.370 |
| 8 | 0.958 | 0.991 | 0.361 | 0.400 | 0.861 | 0.485 | 0.330 | -0.231 |

**Table S2:** Spearman rank correlation coefficients from replicate 2.

|  | **Spearman Rho for relative amount of chlorophyll against RCS** | | **Spearman Rho for relative density of chlorophyll against RCS** | | **Spearman Rho for relative amount of TAG against RCS** | | **Spearman Rho for relative density of TAG against RCS** | |
| --- | --- | --- | --- | --- | --- | --- | --- | --- |
| **Time [days]** | **C2** | **-N2** | **C2** | **-N2** | **C2** | **-N2** | **C2** | **-N2** |
| 0 | 0.963 | 0.962 | 0.201 | -0.028 | 0.656 | 0.562 | 0.237 | 0.238 |
| 1 | 0.960 | 0.987 | 0.031 | 0.360 | 0.707 | 0.836 | 0.235 | 0.420 |
| 2 | 0.962 | 0.993 | 0.016 | 0.471 | 0.661 | 0.767 | -0.129 | 0.371 |
| 3 | 0.968 | 0.995 | 0.230 | 0.416 | 0.662 | 0.572 | -0.104 | 0.084 |
| 4 | 0.974 | 0.991 | 0.206 | 0.491 | 0.741 | 0.333 | -0.055 | -0.208 |
| 5 | 0.970 | 0.992 | 0.334 | 0.444 | 0.606 | 0.419 | -0.013 | -0.249 |
| 6 | 0.967 | 0.989 | 0.348 | 0.383 | 0.642 | 0.578 | 0.220 | -0.286 |
| 7 | 0.964 | 0.989 | 0.283 | 0.401 | 0.815 | 0.570 | 0.212 | -0.232 |
| 8 | 0.955 | 0.991 | 0.269 | 0.425 | 0.866 | 0.608 | 0.187 | -0.312 |

**Table S3:** Fatty acid composition of algal cultures.

| **Fatty acid** | **Mean fatty acid amount (%) in**  **control cultures**  **± SD** | **Mean fatty acid amount (%) in nitrogen starved cultures ± SD** |
| --- | --- | --- |
| **C10:0** | 1.80 ± 0.3 | 0.40 ± 0.4 |
| **C11:0** | 5.86 ± 0.9 | 1.06 ± 0.8 |
| **C12:0** | 0.92 ± 0.1 | 0.19 ± 0.2 |
| **C13:0** | 3.90 ± 0.7 | 0.40 ± 0.4 |
| **C15:0** | 2.42 ± 0.6 | 0.20 ± 0.3 |
| **C16:0** | 29.98 ± 4.8 | 21.74 ± 1.0 |
| **C16:1** | 2.42 ± 1.5 | 4.27 ± 0.3 |
| **C18:0** | 14.16 ± 2.7 | 5.39 ± 1.2 |
| **C18:1** | 8.56 ± 5.6 | 49.14 ± 5.0 |
| **C18:2** | 17.28 ± 9.3 | 6.11 ± 1.0 |
| **C18:3** | 1.26 ± 1.2 | 2.51 ± 1.6 |
| **C20:5** | 3.03 ± 1.4 | 0.55 ± 0.4 |

Means of 2 biological and 3 technical replica each ± S.D.

Literature

1. Lee K., et al. Isolation and screening of microalgae from natural habitats in the midwestern United States of America for biomass and biodiesel sources. Journal of Natural Science, Biology, and Medicine. **5.** 333-339 (2014).
2. Sandmann. M., Lippold. M., Saalfrank. F., Odika. C. P., & Rohn. S. Multi-dimensional single-cell analysis based on fluorescence microscopy and automated image analysis. Anal. Bioanal. Chem. **409**. 4009–4019 (2017).
3. Lichtenthaler HK. Buschmann C. Unit F4.3: Chlorophylls and carotenoids: measurement and characterization by UV-VIS. Curr Protocol Food Anal Chem. 2001;F:F4:F4.3
4. Chen. W., Sommerfeld. M., & Hu. Q. Microwave-assisted Nile red method for in vivo quantification of neutral lipids in microalgae. Bioresource Technology. **102**. 135-141. 10.1016/j.biortech.2010.06.076 (2011).
5. Rumin J. et al. The use of fluorescent Nile red and BODIPY for lipid measurement in microalgae. Biotechnology for Biofuels. **8**. 10.1186/s13068-015-0220-4 (2015).
6. Shapiro HM. *Practical flow cytometry* 197-199 (John Wiley and Sons, 2003).
7. Garz A. et al. Cell-to-cell diversity in a synchronized Chlamydomonas culture as revealed by single-cell analysis. Biophys. J. **103**. 1078-1086 (2012).
8. Rading M. et al. Weak correlation of starch and volume in synchronized photosynthetic cells. Phys Rev E. 91. 10.1103/PhysRevE.91.012711 (2015).
